# Supplementary material for: Adipose tissue and skeletal muscle wasting precede clinical diagnosis of pancreatic cancer
Source: Nat Commun. 2023 Jul 18;14:4317. doi: 10.1038/s41467-023-40024-3 (PMC10354105; doi:10.1038/s41467-023-40024-3)
Supplement: Supplementary file 1 — Supplementary Information [file 41467_2023_40024_MOESM1_ESM.pdf]

Supplementary Table 1. Characteristics of pancreatic cancer cases and matched controls at the time of prediagnosis CT imaging by study site

Characteristic*	DFCI/MGB		KPNC	
	**Cases** (N=228)	**Controls** (N=581)	**Cases** (N=486)	**Controls** (N=1,167)
Age, years**	66.1 (10.9)	65.8 (11.1)	67.6 (9.7)	67.3 (9.9)
Year of CT imaging**				
2003-2010	68 (30)	168 (29)	246 (51)	568 (49)
2011-2017	160 (70)	413 (71)	240 (49)	599 (51)
Intravenous contrast with CT imaging **	161 (71)	409 (70)	402 (83)	1,000 (86)
Female**	106 (46)	272 (47)	234 (48)	555 (48)
Race**				
White	218 (96)	557 (96)	441 (91)	1,046 (90)
Black	10 (4)	24 (4)	45 (9)	121 (10)
Body mass index, kg/m^2***	29.6 (5.9)	28.8 (6.6)	29.2 (6.2)	29.0 (6.4)
Diabetes	51 (22)	128 (22)	142 (29)	240 (21)
Cigarette smoking				
Never	92 (40)	244 (42)	184 (38)	528 (45)
Past	98 (43)	262 (45)	144 (30)	412 (35)
Current	37 (16)	73 (13)	82 (17)	126 (11)
Unknown	1 (0.4)	2 (0.3)	76 (16)	101 (9)
History of alcohol use				
No/Unknown	201 (88)	515 (89)	437 (90)	1,076 (92)
Yes	27 (12)	66 (11)	49 (10)	91 (8)
Prior personal history of cancer before prediagnosis scan	37 (16)	24 (4)	72 (15)	153 (13)
Time from prior cancer to prediagnosis scan, years	14.1 (9.8)	15.5 (9.4)	12.0 (6.5)	10.3 (5.7)
Indication for CT scan				
Symptoms/suspected conditions of the GI system	51 (22)	131 (23)	57 (12)	83 (5)
Symptoms/suspected conditions unrelated to the GI system	62 (27)	239 (41)	102 (21)	468 (27)
Systemic symptoms	16 (7)	54 (9)	49 (10)	106 (9)
New-onset diabetes	0 (0)	0 (0)	4 (1)	6 (1)
Pain	103 (45)	327 (52)	262 (54)	624 (53)
Other/unknown	50 (22)	37 (6)	140 (29)	324 (28)
Age at pancreatic cancer diagnosis, years	67.8 (11.0)	NA	70.1 (9.9)	NA
Pancreatic cancer diagnosis period				
2004-2008	21 (9)	NA	87 (18)	NA
2009-2013	74 (32)	NA	207 (43)	NA
2014-2018	133 (58)	NA	192 (40)	NA
Pancreatic cancer stage at diagnosis				
Non metastatic	132 (58)	NA	238 (49)	NA
Metastatic	93 (41)	NA	233 (48)	NA
Unknown	3 (1)	NA	15 (3)	NA
Time from prediagnosis CT imaging to histopathological diagnosis, months	21.0 (15.9)	NA	23.6 (17.8)	NA
Time from at diagnosis CT imaging to histopathological diagnosis, days****	8 (16)	NA	10 (14)	NA

* Characteristics obtained at the time of the prediagnosis CT imaging study in cases and matched controls. Mean (standard deviation) for continuous variables and N (%) for categorical variables, unless noted otherwise.

** Matching factors

*** Body mass index is unknown for 134 cases and 1 control in DFCI/MGB, and for 62 cases and 119 controls in KPNC.

**** Among 170 DFCI/MGB and 517 KPNC cases with available at diagnosis scan

Abbreviations: DFCI/MGB, Dana-Farber Cancer Institute/Mass General Brigham; KPNC, Kaiser Permanente Northern California

Supplementary Figure 1. Correlation between CA19-9 levels at diagnosis with weight and tissue change in prediagnostic period

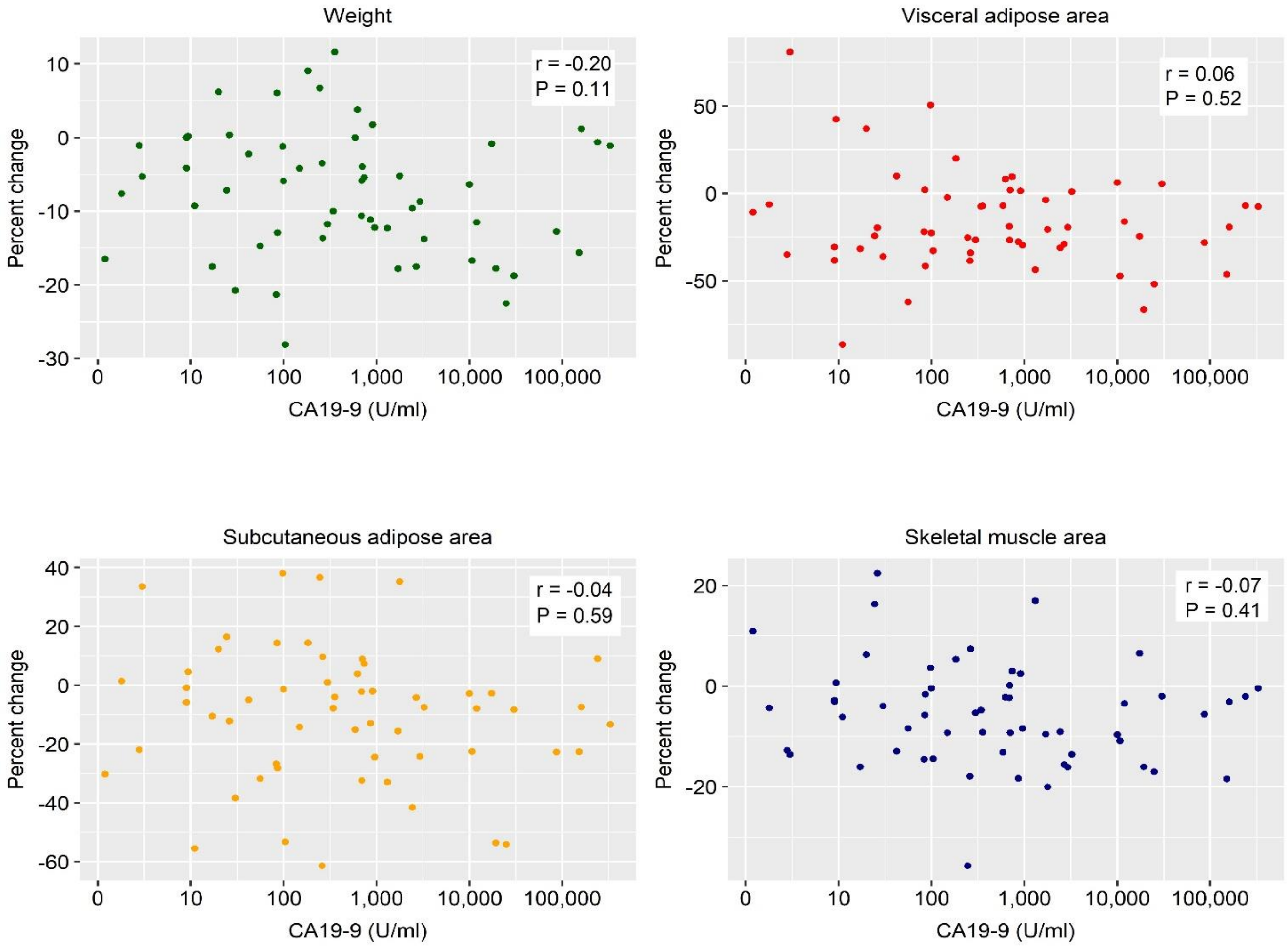

In white boxes are shown Spearman correlation coefficients (r) and corresponding P-values. Source data are provided as a Source Data file.

Supplementary Figure 2. Correlation matrix for percent change in weight and tissue measurements between prediagnosis and at-diagnosis CT scans.

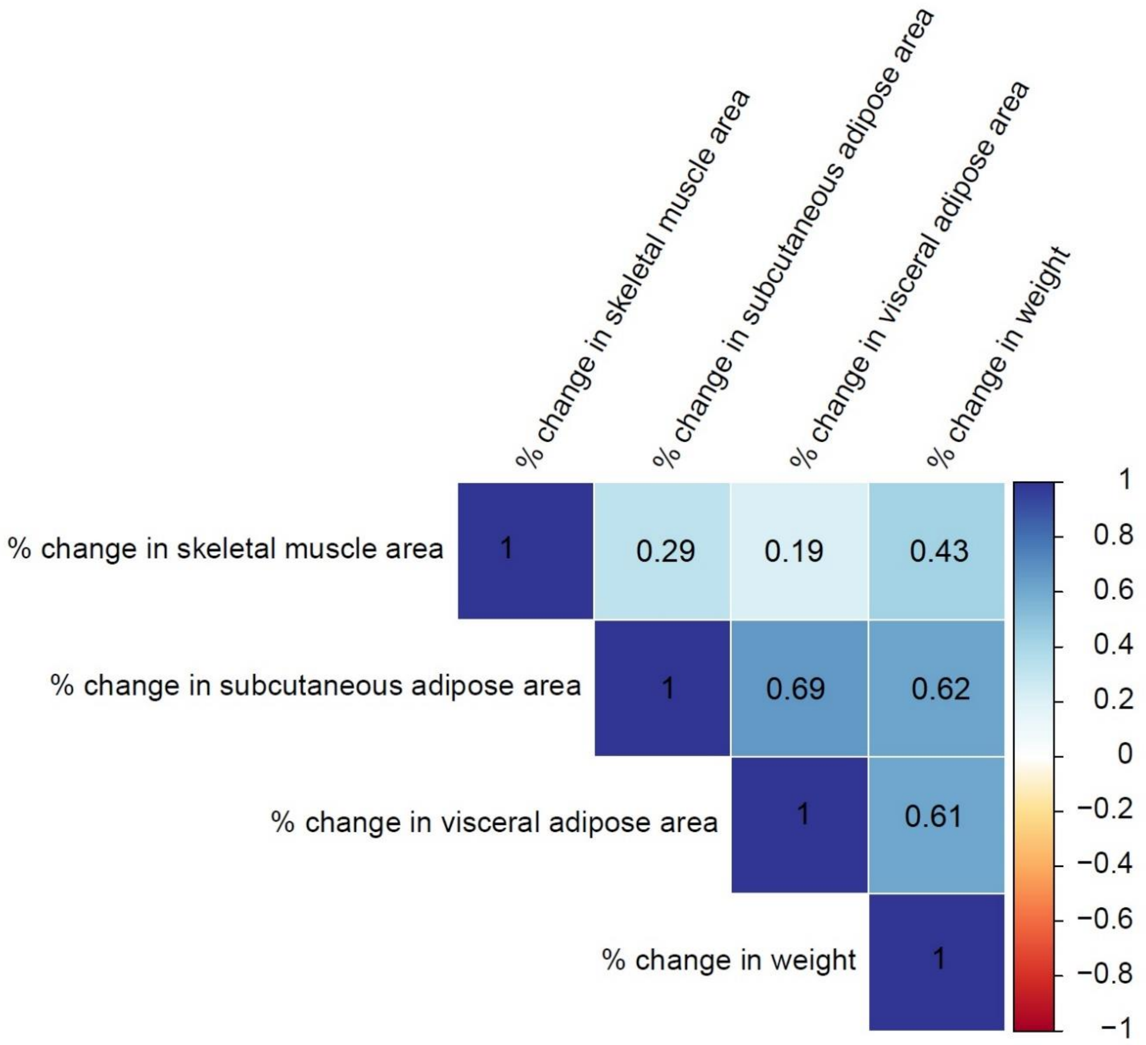

Legend: Color scale represents Spearman correlation coefficients for patients with complete data for tissue and weight change (N=386). Coefficient of correlation is shown for each pair of variables. Source data are provided as a Source Data file.
