# Supplementary material for: Adipose tissue and skeletal muscle wasting precede clinical diagnosis of pancreatic cancer
Source: Nat Commun. 2023 Jul 18;14:4317. doi: 10.1038/s41467-023-40024-3 (PMC10354105; doi:10.1038/s41467-023-40024-3)
Supplement: Supplementary file 3 — Reporting Summary [file 41467_2023_40024_MOESM3_ESM.pdf]

nature portfolio

Corresponding author(s): Brian Wolpin, MD, MPH

Last updated by author(s): May 26, 2023

Reporting Summary

Nature Portfolio wishes to improve the reproducibility of the work that we publish. This form provides structure for consistency and transparency in reporting. For further information on Nature Portfolio policies, see our Editorial Policies and the Editorial Policy Checklist.

Statistics

For all statistical analyses, confirm that the following items are present in the figure legend, table legend, main text, or Methods section.

n/a	Confirmed	
☐	☒	The exact sample size (*n*) for each experimental group/condition, given as a discrete number and unit of measurement
☐	☒	A statement on whether measurements were taken from distinct samples or whether the same sample was measured repeatedly
☐	☒	The statistical test(s) used AND whether they are one- or two-sided<br>*Only common tests should be described solely by name; describe more complex techniques in the Methods section.*
☐	☒	A description of all covariates tested
☐	☒	A description of any assumptions or corrections, such as tests of normality and adjustment for multiple comparisons
☐	☒	A full description of the statistical parameters including central tendency (e.g. means) or other basic estimates (e.g. regression coefficient) AND variation (e.g. standard deviation) or associated estimates of uncertainty (e.g. confidence intervals)
☐	☒	For null hypothesis testing, the test statistic (e.g. *F*, *t*, *r*) with confidence intervals, effect sizes, degrees of freedom and *P* value noted<br>*Give P values as exact values whenever suitable.*
☒	☐	For Bayesian analysis, information on the choice of priors and Markov chain Monte Carlo settings
☒	☐	For hierarchical and complex designs, identification of the appropriate level for tests and full reporting of outcomes
☐	☒	Estimates of effect sizes (e.g. Cohen's *d*, Pearson's *r*), indicating how they were calculated

Our web collection on statistics for biologists contains articles on many of the points above.

Software and code

Policy information about availability of computer code

Data collection	No software has been used for data collection.
Data analysis	Statistical Analysis Software (SAS) version 9.4; R Studio 2022.07.1 Build 554

For manuscripts utilizing custom algorithms or software that are central to the research but not yet described in published literature, software must be made available to editors and reviewers. We strongly encourage code deposition in a community repository (e.g. GitHub). See the Nature Portfolio guidelines for submitting code & software for further information.

Data

Policy information about availability of data

All manuscripts must include a data availability statement. This statement should provide the following information, where applicable:

- Accession codes, unique identifiers, or web links for publicly available datasets
- A description of any restrictions on data availability
- For clinical datasets or third party data, please ensure that the statement adheres to our policy

The raw clinical and imaging data from DFCI/MGB patients are protected and are not available due to data privacy laws. Deidentified data can be obtained from the corresponding author with appropriate IRB approval and inter-institutional data use agreement. The KPNC data are available under restricted access because use was authorized by the KPNC IRB with a waiver of informed consent for this specific study. Access can be obtained by contacting the KPNC research team for review and application for a new IRB approval of a waiver. Processed data used to generate figures has been provided in the Source Data file.

Research involving human participants, their data, or biological material

Policy information about studies with human participants or human data. See also policy information about sex, gender (identity/presentation), and sexual orientation and race, ethnicity and racism.

Reporting on sex and gender	We performed sex-specific analyses of body composition change in period preceding pancreatic cancer diagnosis. Sex was self-reported by participants. We did not perform gender-specific analyses.
Reporting on race, ethnicity, or other socially relevant groupings	Race was self-reported. Only White and Black participants were included in this analysis since reference (or standardized) curves for muscle and adipose tissue measurements for other racial groups have not yet been developed.
Population characteristics	Characteristics of case-control population used in this study are described in detail in Table 1.
Recruitment	Participants (cases and controls) were recruited among patients seen at Dana-Farber Cancer Institute/Mass General Brigham (DFCI/MGB) and Kaiser Permanente Northern California (KPNC) care networks.
Ethics oversight	The current study was approved by Institutional Review Boards at Dana-Farber Cancer Institute, Mass General Brigham, and Kaiser Permanente Northern California.

Note that full information on the approval of the study protocol must also be provided in the manuscript.

Field-specific reporting

Please select the one below that is the best fit for your research. If you are not sure, read the appropriate sections before making your selection.

☒ Life sciences ☐ Behavioural & social sciences ☐ Ecological, evolutionary & environmental sciences

For a reference copy of the document with all sections, see nature.com/documents/nr-reporting-summary-flat.pdf

Life sciences study design

All studies must disclose on these points even when the disclosure is negative.

Sample size	Population consisted of 714 pancreatic cancer cases and 1,748 matched controls. Number of cases was limited by availability of CT imaging in prediagnostic period (2 months to 5 years before diagnosis) and other exclusion criteria (indication for prediagnosis CT scan related to PDAC diagnosis, history of another cancer with unknown date of diagnosis or diagnosis <5 years before prediagnosis CT scan, and age, sex, and race-specific reference curves for tissue areas not available). Cases were then matched with 1 to 3 controls without pancreatic cancer with available CT imaging.
Data exclusions	Exclusion criteria for case-control population are described above. No data was excluded from the analysis.
Replication	No external dataset was available for validation.
Randomization	Not applicable. This study was observational and did not have experimental or treatment groups.
Blinding	Investigators were blinded to case-control status during image analysis and quantification of adipose and skeletal muscle tissues.

Reporting for specific materials, systems and methods

We require information from authors about some types of materials, experimental systems and methods used in many studies. Here, indicate whether each material, system or method listed is relevant to your study. If you are not sure if a list item applies to your research, read the appropriate section before selecting a response.

Materials & experimental systems

n/a	Involved in the study	
☒	☐	Antibodies
☒	☐	Eukaryotic cell lines
☒	☐	Palaeontology and archaeology
☒	☐	Animals and other organisms
☐	☒	Clinical data
☒	☐	Dual use research of concern
☒	☐	Plants

Methods

n/a	Involved in the study	
☒	☐	ChIP-seq
☒	☐	Flow cytometry
☒	☐	MRI-based neuroimaging

Clinical data

Policy information about clinical studies

All manuscripts should comply with the ICMJE guidelines for publication of clinical research and a completed CONSORT checklist must be included with all submissions.

Clinical trial registration	N/A (not a clinical trial)
Study protocol	N/A (not a clinical trial)
Data collection	CT imaging, clinical and demographic data was collected from 2003 to 2017
Outcomes	N/A (not a clinical trial)
